# Effectiveness and equity of sugar-sweetened beverage taxation

**DOI:** 10.1371/journal.pmed.1002327

**Published:** 2017-06-27

**Authors:** Sanjay Basu, Kristine Madsen

**Affiliations:** 1 Department of Medicine, Stanford University, Palo Alto, California, United States of America; 2 School of Public Health, University of California Berkeley, Berkeley, California, United States of America

## Abstract

Sanjay Basu and Kristine Madsen discuss the effects of taxes on sugar-sweetened beverages in both Australia and Berkeley, USA.

Given the global epidemics of obesity, type 2 diabetes, and associated cardiovascular disease, numerous strategies are being discussed and implemented to reduce the risk of excess calorie intake and the adverse metabolic effects of unhealthy sugar consumption. While individual-level interventions such as educational and behavioral modification strategies have been extensively researched, they remain difficult to effectively implement in large populations. Hence, fiscal and regulatory interventions such as sugar-sweetened beverage (SSB) taxation have been widely discussed. SSB taxation is of particular interest given consistent evidence associating SSB consumption with elevated risk of metabolic and cardiovascular disease and the lack of any known positive value of SSBs for nutrition or human health. Moreover, SSBs can be isolated from other foods easily, there are negative externalities of SSB consumption in terms of excess healthcare and disability costs, and strong evidence exists to indicate that taxation has been an effective means for reducing consumption of other unhealthy products such as tobacco [1–4].

Contributing to the debate on the public health case for taxing SSBs, 2 recent articles in *PLOS Medicine* [5,6] add to the evidence concerning SSB taxation, which has mostly come from Mexico after the country implemented an approximately 10% tax on SSBs in 2014 [7–10]. The new articles address major questions about the effects of SSB taxation: Are SSB taxes passed through from retailers to consumers, and, if so, are the price increases sufficient to reduce SSB consumption? Does substitution of SSBs by other beverages or foods neutralize the benefits of the tax; and does the tax harm retailers or consumers, particularly consumers of low socioeconomic status (SES)?

## SSB data from Berkeley, California

In the first of the 2 articles, Barry Popkin and colleagues performed a study of prices, sales, consumer spending, and beverage consumption before and after a penny-per-ounce tax on SSBs that was implemented starting in March 2015 in Berkeley, California, United States [5]. The authors made an extensive effort to triangulate data from stores, sales databases, and telephone surveys of adults in Berkeley. Importantly, sales data analyses included adjacent comparison cities that did not institute a tax, helping to isolate the effects of the tax from macroeconomic conditions that can affect beverage purchases over time, preexisting trends, regional media campaigns that both favored and opposed the tax, and other social and cultural influences on SSB sales across the region.

Popkin and colleagues’ results, consistent with a previous report [11], reveal that the SSB tax in Berkeley was generally passed through to consumers; notably, pass-through was substantial at the major chain supermarkets at which most SSBs are purchased but not at small venues at which fewer SSBs are sold. Pass-through of the tax was also more consistent for carbonated than noncarbonated SSBs, as was the case in Mexico [7], suggesting that guidelines for retailers that help clarify which products to apply the tax to may be important for effective and consistent tax implementation. Second, sales of taxed SSBs in Berkeley fell, and sales of untaxed beverages (water, teas, milk) rose, as compared to adjacent cities without the tax. Whether Berkeley residents purchased SSBs in adjacent cities was not clear; participant surveys suggested they did not, while sales data showed increased purchase in adjacent cities, which could have been from residents of those cities or from a mix of those residents and Berkeley residents. Importantly, the tax did not produce overall higher grocery bills or revenue declines for retailers, contrary to a common argument against SSB taxation. Hence, the tax was largely effective at influencing sales in the manner it was intended to, which is particularly notable because Berkeley already had lower consumption and higher average income than most US cities—potentially blunting the effects of the tax in Berkeley as compared to other locations.

The results of the sales data in Popkin and colleagues’ study are clear, but it is important to contextualize the less unequivocal consumption data. No control group data were available for the consumption data; hence, the net calorie change estimate in the study does not reflect the impact of the tax per se, but it includes effects of both the tax and regional changes in non-SSB beverage consumption unrelated to the tax. The net decrease in SSB consumption (not just sales) in the study was large, but low sample size left the study underpowered to study SSB consumption rather than sales. Nevertheless, a larger study in Berkeley with 2 control groups showed significant SSB consumption declines of similar magnitude but greater precision [12].

## Simulations of the equity of taxation in Australia

In the second *PLOS Medicine* article, Anita Lal and colleagues projected changes in SSB consumption across the SES spectrum using data from Australian health surveys [6]. This study addresses the question of whether a hypothetical 20% SSB tax would be regressive (disproportionately burdening low-SES populations), even when accounting for health benefits and healthcare cost savings.

The study used simulations to project tax effects by examining the potential reduction in SSB consumption and a range of possible beverages substituted for SSBs, using estimates of price elasticity that were consistent with the experience in Mexico, Berkeley, and elsewhere. The simulation estimated changes in body mass index based on changes in caloric intake and associated changes in clinical endpoints associated with obesity. The simulation is unique in specifically examining the distribution of financial impacts across different SES groups, both in terms of out-of-pocket costs incurred from the tax and in terms of healthcare costs saved and health gains from future disease averted.

Although there are inherent uncertainties and assumptions in such simulations, they help systematically organize our current evidence to inform decisions for which randomized trials or standard epidemiological study designs are rarely feasible. Lal and colleagues’ simulation provides reassurance that, under a broad range of plausible SSB tax effects, the regressivity of the tax in terms of out-of-pocket costs to consumers would likely be far exceeded by benefits in terms of healthcare costs averted, benefiting the lowest-SES strata overall, even after accounting for the lower valuation of future dollars relative to present dollars (discounting) and the market inefficiencies inherently produced by taxation (deadweight loss).

## Implications for public health policy

Further data will emerge from other cities and countries now that SSB taxation has passed in a number of other jurisdictions (including the United Kingdom and, in the US, the cities of Albany, Boulder, Chicago, Oakland, Philadelphia, and San Francisco) and is being considered in other countries, including Australia, India, and South Africa, despite intimidation faced by tax advocates in some locales [13]. As proposed legislation unfolds, research addressing the central question of how some jurisdictions but not others successfully pass SSB tax legislation would extend the evidence gathered from the Popkin et al. study. While cities like Berkeley are characterized as unique outliers for the passage of public health legislation, the highest-risk populations are not in such cities. Notably, SSB tax advocates in Philadelphia passed an SSB tax by framing the legislation as a funder of child education, not as a public health measure [12]. The Berkeley measure, in its first year, raised over $1.4 million for the city, about $12 per capita [1].

The revenue effects of SSB taxation can render it far more attractive to policymakers than some alternatives, despite the political pressures to avoid a fiscal or regulatory approach to intervention. Given the accumulation of evidence from Mexico, Berkeley, and simulation studies of multiple locales, SSB taxation has strong evidence suggesting that it is likely to reach broader populations, and with likely greater effectiveness, than individual behavior-change interventions or interventions based entirely within the healthcare system. The key task that remains, as was the case with tobacco [14], is to identify and study a suite of supportive measures that may be necessary to achieve sustained reductions in SSB consumption. Workplace sales bans, SSB warning labels (like “black box” warnings on cigarette packages), restrictions on sales to youth, and similar measures can be considered complements to taxation and require further analysis to understand their synergy with SSB taxation.

## Abbreviations

SES: socioeconomic status
SSB: sugar-sweetened beverage
